# Rewired DDR-TGF-β-β-catenin-PD-L1 axis accelerates progression and shapes therapy in human papillomavirus-driven cancer

**DOI:** 10.3389/fimmu.2026.1907171

**Published:** 2026-07-22

**Authors:** Wei Liu, Shanmei Chen, Liwei Wang, Hua Xiang, Siyi Tao

**Affiliations:** 1 Hunan Cancer Hospital, Affiliated Hospital of Xiangya Medical School, Central South University, Changsha, Hunan, China; 2 Shaoyang Central Hospital, Shaoyang, Hunan, China; 3 Key Laboratory of Carcinogenesis of Ministry of Health, Central South University, Changsha, Hunan, China; 4 Second Xiangya Hospital, Central South University, Changsha, Hunan, China

**Keywords:** β-catenin, cancer, DNA damage response, FAT4, HPV, PD-L1, TGF-β

## Abstract

Understanding Human papillomavirus (HPV) oncogenic mechanisms is essential for developing preventive and therapeutic strategies and overcoming therapy resistance in HPV-related cancers. These challenges may arise from the ability of high-risk HPV to subvert host tumor suppressors such as p53 and Rb, and to drive oncogenesis through homologous recombination deficiency (HRD) and multi-network dysregulation. Mechanistically, HPV exerts context-dependent effects on the host DNA damage response (DDR). During episomal replication, E6/E7 activate DDR and recruit BRCA1/RAD51 to replication foci to support viral replication without significantly compromising host HR repair. Upon viral integration, however, sustained E6/E7 expression drives HRD and shifts DNA repair toward error-prone end joining, generating genomic instability that fuels malignant transformation. These alterations are most clearly established in cervical cancer, whereas evidence in HPV-positive non-cervical cancer is more variable and requires further context-specific validation. In parallel, HPV E6/E7 antagonize transforming growth factor-β (TGF-β)-mediated tumor suppression and, potentially through FAT Atypical Cadherin 4 (FAT4) down-regulation, engage Wnt/β-catenin signaling. The resultant elevation of nuclear β-catenin induces programmed death-ligand 1 (PD-L1) expression promotes immune evasion, stemness, and invasiveness. Of note, while the DDR-TGF-β-β-catenin-PD-L1 axis is backed by substantial evidence in HPV-related cancers, certain connections within this pathway are extrapolated from non-HPV models or general pathway biology and are explicitly denoted as such in the main text. With residual p53 activity, HRD may confer initial sensitivity to DNA-damaging agents, but resistance frequently develops—a pattern reminiscent of the initial response followed by acquired resistance observed with immunotherapies in HPV-related cancers. Integrating these mechanistic insights, we propose ablative therapies (e.g., ablation, photodynamic therapy, surgery) for cervical intraepithelial neoplasia (CIN), and for advanced or resistant disease, a synthetic-lethality framework combining genotoxic therapies with DDR inhibitors, targeting DDR-TGF-β-β-catenin-PD-L1 axis, and antiviral approaches. The proposed therapeutic strategies, however, should be interpreted with caution, as their evidence base varies across tumor types and warrants further investigation.

## Background

Human papillomavirus (HPV) is a circular double-stranded DNA virus of approximately 8 kilobases. Over 200 HPV types infect human skin or mucosal tissues (1). Based on oncogenic potential, HPVs are classified as low- or high-risk (cancer-associated) type. Low risk types cause anogenital and oral warts or benign skin lesions. High-risk types, including HPV16, 18, 31, 33, 35, 39, 45, 51, 52, 56, 58, 59, and 68, cause anogenital and oropharyngeal cancers (2). HPV transmission occurs through direct skin or mucosal contact, predominantly through sexual activities (2). HPV vaccination remains a cornerstone of prevention. The quadrivalent vaccine, which targets HPV 6, 11, 16, and 18, and the 9-valent vaccine, which targets additional types, show high efficacy. By 2022, 45 countries had expanded HPV vaccination to boys, representing a critical step toward eliminating cervical cancer and reducing other HPV-associated cancers.

Despite vaccination, HPV-associated cancers remain a major global health burden. Among infectious carcinogens, HPV ranks second in population attributable fraction (31.1%) (3). Under active surveillance, stepwise progression from CIN1 to invasive cancer still occurs in a subset of cases, highlighting a mechanistic gap that precludes progression-risk stratification, precise blockade, and fertility preservation. Loop Electrosurgical Excision Procedure (LEEP), conization and ablation achieve high regression rates but cause irreversible cervical damage. Photodynamic therapy (PDT) is mechanism-based and tissue-preserving, yet has lower regression and HPV clearance rate, limited penetration, and higher recurrence. Imiquimod shares similar limitations. A summary of current CIN therapies and their effects is presented in **Table 1** (4–12). High-risk HPVs account for approximately 5% of cancers worldwide. Persistent high-risk HPV causes >99% of cervical cancers, 88% of anal cancers, 70% of oropharyngeal and vaginal/vulvar cancers, and approximately 50% of penile cancers (13). The global incidence and mortality of HPV 16 and 18 caused cervical cancer are projected to rise, with few prospects for near-term control. HPV 16-positive oropharyngeal squamous cell carcinoma (OPSCC) has markedly increased, particularly in developed countries. Although HPV-positive OPSCC confers a favorable prognosis, nodal metastasis reduces survival by approximately 50%.

**Table 1:** Summary of different management strategies for Cervical Intraepithelial Neoplasia (CIN).

Management strategy	HPV clearance rate	Histologic regression rate	Progression/recurrent disease rate
Observation / Active Surveillance	80-90% at 24 months	60% CIN 1 and 55% CIN 2 naturally regress	11% CIN 1 and 19% CIN 2 progress, but only 0.3% of them progress into cervical cancer (4)
Surgical Excision (Conization / LEEP)	LEEP: 64.3% at 6 months;	84% complete histologic remission	residual rate of HPV was 22.7%, imargin negative 0.5% recurrent. margin positive 18% recurrent (5).
	LEEP 87.5% at 12 months	histologic cure 95.2% (6)	margin negative 1,8% recurrent. margin positive 15.7% recurrent.
	55.3% versus 66% of surgery	55% reduction to ≤ CIN 1 versus 93% of surgery (7)	
Topical Agent (Imiquimod)	22% HPV clearance with imiquimod versus 88% with surgery in residual or recurrent CIN	33% regression with imiquimod versus 100% with surgery in residual or recurrent CIN (8)	18.8% residual rate with CIN
Ablative Therapy (Cryotherapy)	HR-HPV clearance rate: 80.4% HPV clearance 72.4% at 6 months and 81.0% at 12 months	Regression CIN 2+: 76.2%; CIN 1: 90.3% for thermal ablation (9) The effective rate at 6 months 96.8% (10).	recurrence rate at 6-12 months was 2.0%. for ultrasound ablation
photodynamic therapy (PDT)	PDT's 78.6 % VS. LEEP's 60.3 %	PDT's 83.3 % VS. LEEP's 64.7 % (11),	recurrence rate at 24 months was 7.1% (12)
	HPV clearance rate during the 6-month was 51.8 %	total regression rate was 89.3 %	

TGF-β acts as a tumor suppressor during initial HPV infection but undergoes a functional switch to a tumor-promoting state that facilitates viral persistence and immune evasion, a phenomenon termed the TGF-β paradox (14). Toll-like receptor 7 agonist imiquimod has been shown to enhance HPV clearance through stimulating innate and adaptive immunity, antigen presentation, cytotoxic T lymphocyte activation, and apoptosis; however, human urine stem cell-derived TGF-β1 ameliorates imiquimod-induced psoriasis through Janus kinase 2 inhibition and a M1-M2 macrophage switch (15). Therefore, the regulatory connection between TGF-β1 and imiquimod requires further study. HPV DNA integration frequently disrupts the viral E2 gene, leading to constitutive overexpression of *E6* and *E7* oncogenes, which inactivate p53 and pRb, respectively, thereby driving proliferation and impairing apoptosis. Benzo[a]pyrene diol epoxide induces DNA double-strand breaks (DSBs) at regions that overlap with HPV integration sites in the host genome. This may explain how tobacco smoke cooperates with HPV infection to cause cancer (16). HPV-induced chronic reactive oxygen species (ROS) that promotes mutagenic DSBs and carcinogenesis. PDT generates high-flux ROS upon light activation of photosensitizers which preferentially accumulate in HPV-infected and tumor cells, leading to selective cytotoxicity, vascular damage, and immune response. When no other risk factors are present, HPV-positive oropharyngeal cancers respond better to treatment than HPV-negative ones (17). Barcellos-Hoff et al. proposed an insightful framework to explain and translate these observations and other preclinical evidence: HPV-mediated loss or inhibition of the TGF-β signaling shifts DNA repair from homologous recombination (HR) and non-homologous end joining (NHEJ) toward alternative end-joining repair independent of a sister chromatid template, thereby sensitizing cancer cells to genotoxic treatment (18, 19). For instance, TGF-β signaling blockade increases the sensitivity of lung, brain, ovarian, breast, and head and neck cancers to chemotherapy- or irradiation-induced DNA damage in preclinical cell and xenograft models (20).

The Wnt/β-catenin pathway has been implicated in the DDR, and HPV E6/E7 may engage this pathway through mechanisms that remain to be fully defined. In support of a link between these pathways, nuclear α-catenin, β-catenin, and actin have been associated with DNA repair processes, and β-catenin has been reported to regulate DNA ligase IV (21). Additionally, Glycogen Synthase Kinase 3 Beta (GSK3β)-mediated phosphorylation of p53-Binding Protein 1 (53BP1) at Thr334 has been shown to influence DNA repair pathway choice (22). HPV E6/E7 have been reported to upregulate DNA topoisomerase I—which functions as a DNA repair mediator—and to activate cGAS, with downstream effects that may promote PD-L1-mediated immune evasion (23). HPV infection has also been linked to upregulation of DNA repair proteins including BRCA1, RAD51, and FANCA/FANCI. However, E6/E7 have been shown to disrupt HR, an effect attributed to the promotion of error-prone repair and impaired RAD51 localization to DSBs (24). HR proteins, including NBS1, WRN, BRCA1, RAD51, and TOPBP1, are recruited to viral replication foci, a process that may contribute to genomic instability (25). Serpin Family B Member 3 (SERPINB3) stabilizes the FANCD2-FANCI complex via USP1-mediated deubiquitination. Of note, SERPINB3 knockdown sensitizes HPV-negative head and neck cancer cells to cisplatin (26), suggesting a potential but unconfirmed relevance to HPV-positive contexts. Collectively, these observations suggest potential therapeutic avenues to enhance tumor sensitivity to genotoxic therapies, though mechanistic validation in HPV-positive settings remains incomplete.

Notably, the translational potential of targeting HPV-positive carcinomas is further reinforced by the role of E6/E7 in disrupting the β-catenin/PD-L1 and DNA repair pathways. The Wnt/β-catenin pathway has been implicated in the DDR, and HPV E6/E7 may engage this pathway through mechanisms that remain to be fully defined. For instance, nuclear α-catenin, β-catenin, and actin are associated with DDR, and β-catenin has been reported to DNA ligase IV (21). Glycogen synthase kinase-3 beta (GSK3β)-mediated phosphorylation of 53BP1 at Thr334 has been shown to influence the DNA repair pathway choice (22). Evidence also indicates that HPV E6/E7 upregulate DNA topoisomerase I, which functions as a DNA repair mediator and activates cyclic GMP–AMP synthase (cGAS), thereby promoting PD-L1-mediated immune evasion (23). HPV infection has also been linked to upregulation of DNA repair proteins, including BRCA1, RAD51, and Fanconi anemia complementation group A and group I (FANCA/FANCI). However, E6/E7 have been shown to disrupt HR by promoting error-prone template-free DNA repair and impairing RAD51 localization to DSBs (24). HPV E6/E7 proteins induce host DNA damage, while HR proteins, including Nijmegen Breakage Syndrome 1 (NBS1), WRN, BRCA1, RAD51, and TOPBP1, are preferentially co-opted to support viral replication, a process that may contribute to genomic instability (25). These tumors usually retain residual p53 function to induce apoptosis despite E6-mediated suppression. Moreover, Serpin Family B Member 3 (SERPINB3) supports ubiquitin-specific peptidase 1-mediated deubiquitination of the FANCD2–FANCI complex; its suppression by HPV or knockdown inhibits DNA repair, and SERPINB3 knockdown sensitizes HPV-negative head and neck cancer cells and xenografts to cisplatin (26). Collectively, these observations suggest potential therapeutic avenues to enhance tumor sensitivity to genotoxic therapies, though mechanistic validation in HPV-positive settings remains incomplete.

**Figure 1:**
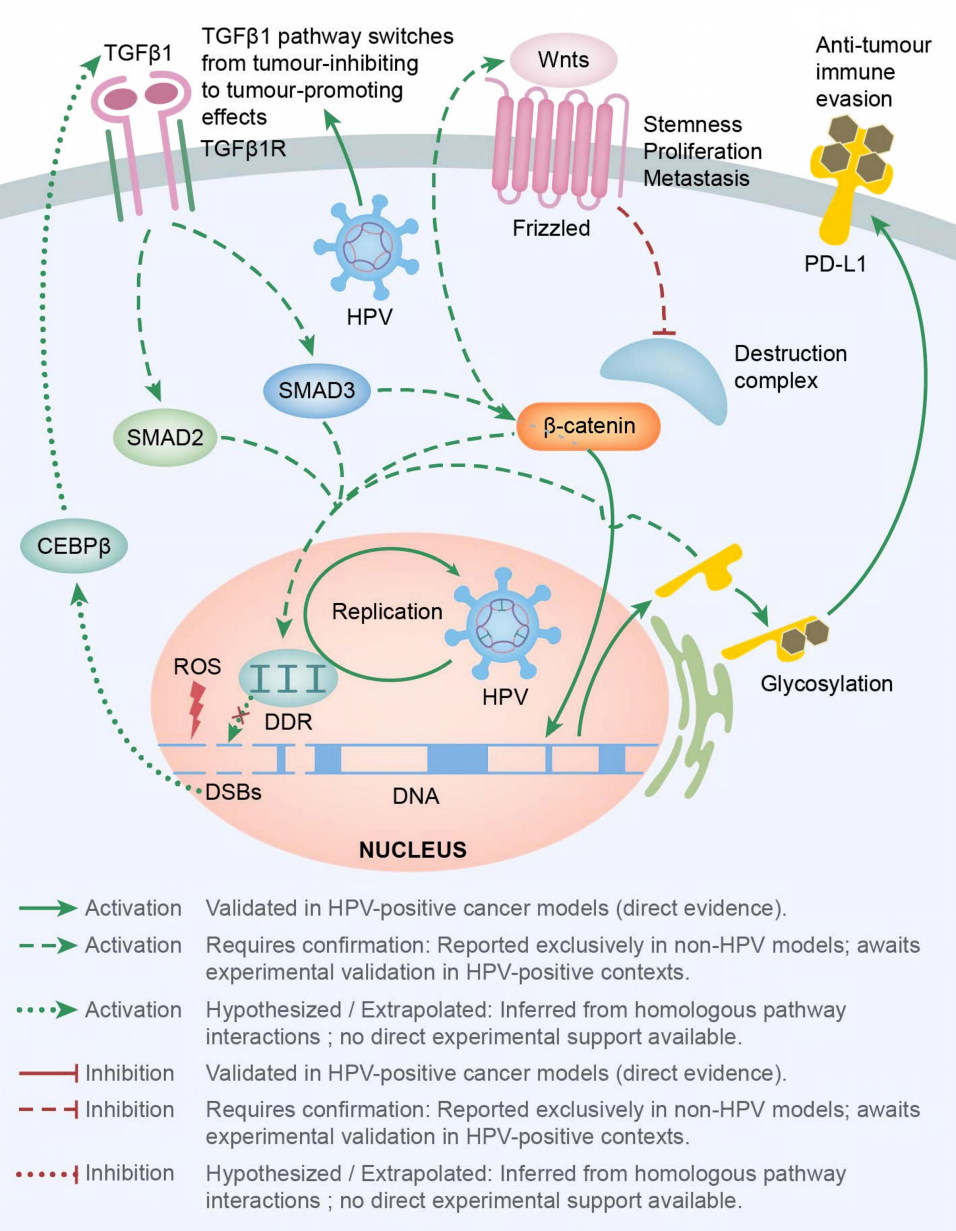
HPV rewires the DNA damage response and TGF-β/β-catenin/PD-L1 axis to drive malignant progression. HPV-induced chronic reactive oxygen species (ROS) may promote DSBs that drive mutagenesis and carcinogenesis. ROS appear to activate C/EBPβ, which elevates TGF-β1 expression. HPV infection subverts TGF-β1 from tumor-suppressive to tumor-promoting effects, stimulating β-catenin nuclear activity and Wnt expression to amplify Wnt/β-catenin signaling (13). Elevated Wnt/β-catenin signaling promotes stemness and proliferation, thereby contributing to metastatic dissemination. Nuclear β-catenin, as validated in several independent studies, activates STT3A transcription and induces PD-L1 expression; STT3A promotes PD-L1 glycosylation and membrane localization, thereby facilitating immune evasion (98). DSBs activate the DDR pathway, which meantime activate the TGF-β/β-catenin/PD-L1 axis to coordinate DNA repair, whereas HPV co-opts key DDR factors to support viral replication (25). Collectively, oncogenic rewired DDR-TGF-β-β-catenin-PD-L1 axis contributes to malignant progression of HPV-positive cells. A discreet discrimination is made throughout the figure among three tiers of evidence: experimentally confirmed in HPV-positive cervical, HNSCC, and anogenital cancers; supported by non-HPV model systems; and postulated from canonical pathway extrapolation.

Recent findings indicate that mutually-connected TGF-β, β-catenin, and PD-L1 pathways are differentially linked to DDR and synthetic lethality (See **Figure 1**). **Table 2** systematically synthesizes the current landscape of mechanistic evidence and critical knowledge gaps regarding the TGF-β, β-catenin, DDR, and PD-L1 axes in HPV-related cancers (133, 134). It highlights that while the DDR-PD-L1 and DDR-TGF-β connections are experimentally established in non-HPV models, they remain unverified in HPV-positive cancers. Moreover, the cross-talk involving TGF-β-β-catenin and β-catenin-DDR remains largely hypothetical, which underscores urgent priorities for future mechanistic and translational investigations.

**Table 2:** Emerging evidence on TGF-β, β-catenin, PD-L1, and DDR interactions in HPV-related cancers.

Pathway/molecule	Key findings (emerging evidence)	Experimental model(s)	Therapeutic implications
TGF-β and PD-L1	Bintrafusp alfa (TGF-β/PD-L1 bispecific) + PDS0101 vaccine achieved 62.5% ORR in ICI-naive HPV-16+ patients; PRGN-2009 vaccine + bintrafusp alfa achieved mOS 24.6 months in ICI-resistant patients.	Phase II trials (NCT04246489, NCT04489940); HPV- 16+ cervical and HNSCC	Dual TGF-B/PD-L1 blockade promising in HPV+ subsets, especially with therapeutic vaccines
ß-catenin and PD-L1	FAT4 overexpression binds ß-catenin, promotes its degradation, and suppresses PD-L1/STT3A transcription, enhancing CTL activity in cervical cancer models.	Cervical cancer cell lines (ME180, SiHa, Caski, U14); mouse xenografts	Targeting FAT4/β-catenin/STT3/PD-L1 axis may offer novel immunotherapy combinations for cervical cancer
DDR and PD-L1	HPV E6/E7 - TOP1 - cGAS-STING-NF-kB - PD-L1 overexpression; TOP1 inhibitors block this cascade. Axis well-established in non-HPV models but HPV- specific validation incomplete.	HPV+ cervical cancer cell lines (SiHa, CaSki); TOP1 inhibitors; xenografts	TOP1 inhibitors may reverse PD-L1-mediated immune evasion in HPV+ tumors
TGF-β and DDR	TGF-β modulates DNA repair genes (BRCA1, ATM, RAD51). Preclinically confirmed in non-HPV models that TGF-B loss shifts repair to error-prone pathways, conferring PARP inhibitor sensitivity.	Non-HPV cancer models; normal fibroblast systems	TGF-β inhibition may synergize with DDR-targeted agents in HPV+ tumors
TGF-β-β-catenin	HPV E6/E7 activate both TGF-β and Wnt/β-catenin pathways; mechanistic crosstalk plausible but HPV- specific evidence scarce.	Limited to non-HPV or transgenic models	Combined TGF-β and ß-catenin targeting may have therapeutic potential
ß-catenin-DDR	ATM/ATR can phosphorylate β-catenin and modulate its stability. Impaired DDR may synergize with β- catenin to enhance CSC properties.	Limited to non-HPV cancer types; no HPV-specific data	Biologically plausible but hypothetical in HPV-driven cancers

CTL, cytotoxic T lymphocyte; ICI, immune checkpoint inhibitor; mOS, median overall survival; ORR, objective response rate; TOP1, topoisomerase I.

To construct this narrative/perspective review, we searched PubMed/MEDLINE and Web of Science (2017–2026) for (HPV OR human papillomavirus) AND (cervical cancer OR HPV-associated cancer) AND (DNA damage response OR TGF-β OR β-catenin OR PD-L1). We supplemented this with foundational pre-2017 studies to ensure mechanistic continuity. Additionally, we leveraged key advances from the broader cancer research landscape in which DDR, TGF-β,β-catenin, and PD-L1 crosstalk has been more extensively characterized, to inform our understanding of these pathways in the HPV context. Inclusion criteria were original research/reviews on mechanistic crosstalk among the four pathways in HPV-related cancers, preclinical studies with translational endpoints, and clinical trials reporting therapeutic outcomes. We excluded non-English articles, case reports, conference abstracts, and studies without therapeutic/mechanistic relevance. The initial search retrieved 521 records; after title/abstract screening, we retained 154 full-text articles for detailed assessment. We prioritized direct mechanistic connections and high-impact translational findings, and based on consensus among authors, we formulated a mechanistic model and explored its potential therapeutic implications. Based on HPV pathogenesis, we propose the use of surgery, ablation, PDT, imiquimod, or other immunotherapy-based strategies for CIN, and a synthetic-lethality approach for cancer. This review brings together mechanistic insights, discusses translational advances, and highlights future research directions.

## Integral role of the DDR in HPV life cycle and oncogenesis

The HPV life cycle has three keratinocyte differentiation-linked phases: establishment in basal keratinocytes (50–100 copies per cell), maintenance in undifferentiated cells via E2-mediated episome tethering, and productive replication once differentiation begins. The last phase amplifies the genome extensively (100 to >1,000 copies per cell) in a G2-arrested state, hijacking the host DDR to replicate without entry into the host S phase (27). It also drives late capsid protein production, assembles virions, and releases them from differentiated epithelial cells. The viral genome usually persists as an episome, but it can integrate into the host genome through DDR pathways after E6/E7-induced DSBs (28, 29).

DNA damage triggers a series of phosphorylation events that drive the DDR. Three types of DDR components act in sequence: sensors detect the damage, transducers amplify and pass the signal along, and effectors carry out the repair. Different sensor complexes handle different types of damage. DSBs are recognized by the MRE11/RAD50/NBS1 complex (MRN complex), composed of Meiotic Recombination 11 Homolog A (MRE11), RAD50, and NBS1. Single-stranded DNA breaks (SSBs) are detected and bound by Replication Protein A (RPA) and the RAD9–RAD1–HUS1 complex (9-1–1 complex). These complexes then recruit transducer kinases from the phosphoinositide 3-kinase-related kinase family: Ataxia Telangiectasia Mutated (ATM) and DNA-Dependent Protein Kinase Catalytic Subunit (DNA-PKcs), activated at DSB loci and Ataxia Telangiectasia and Rad3-Related (ATR) kinase localizes to SSB loci. One of the earliest events following DSB generation is the phosphorylation of the histone variant H2A.X to form γH2A.X, which marks DSBs and promotes recruitment of repair machinery. ATM and ATR then activate their respective downstream effectors, Checkpoint Kinase 2 (CHK2) and Checkpoint Kinase 1 (CHK1), through phosphorylation. These effectors pass signals to key regulators of the DDR (e.g.,p53) and core cell cycle regulators (e.g., cell division cycle 25 phosphatases). Through this network cellular fate is determined: cells either undergo cell cycle arrest to allow repair, senescence, or apoptosis if the damage is beyond repair (30–32).

**Figure 2:**
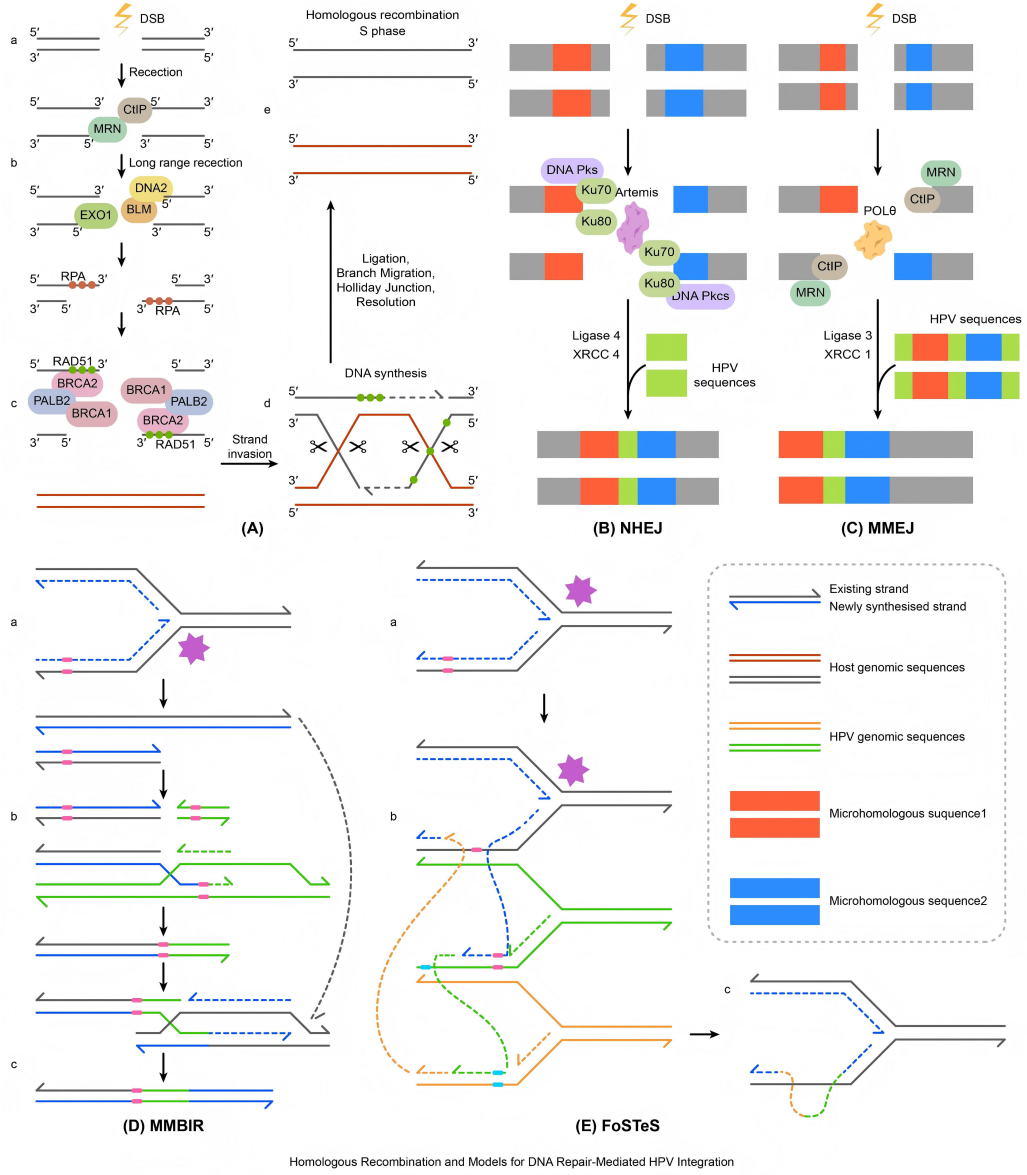
Homologous recombination and four models for DNA-repair-mediated HPV integration. **(A)** Homologous recombination (HR). **(a)** MRN (MRE11-RAD50-NBS1) senses DSBs. **(b)** MRE11-CtIP initiates short-range resection, then BLM-DNA2 or EXO1 extends it to generate RPA-coated ssDNA overhangs. **(c)** BRCA1-PALB2-BRCA2 replaces RPA with RAD51 on ssDNA. **(d)** RAD51 filaments invade a sister chromatid, forming a D-loop to prime high-fidelity DNA synthesis. **(e)** After ligation, branch migration yields double Holliday junctions, which are cleaved by GEN1 into crossover or non-crossover products. **(B)** Non-homologous end joining (NHEJ). **(a)** A DSB forms in the host genome. **(b)** Ku70/Ku80 and DNA-PKcs bind to broken ends independent of homology. **(c)** XRCC4-DNA ligase 4 seals ends, often introducing small indels. **(C)** Microhomology-mediated end joining (MMEJ). **(a)** A DSB forms. **(b)** MRN-CtIP resects ends 5′→3′ to expose microhomology. **(c)** Host microhomology anneals to complementary HPV sequences; XPF-ERCC1 removes non-homologous 3′ overhangs, and DNA polymerase θ fills gaps. **(d)** DNA ligase 3-XRCC1 seals nicks, causing deletions or rearrangements at the host site. **(D)** Microhomology-mediated break-induced replication (MMBIR). **(a)** A single-ended DSB arises from breakage or fork collapse. **(b)** 5′→3′ resection generates a 3′ overhang exposing microhomology. **(c)** The overhang invades HPV microhomology and primes DNA synthesis. **(d)** The newly extended strand disengages and re-anneals to the original template. **(e)** Repeated cycles generate host-HPV chimeras and drive rearrangements or integration. **(E)** Fork-stalling and template-switching (FoSTeS). **(a)** Replication fork stalls at host lesions or breaks. **(b)** The stalled fork disengages and switches to HPV DNA as an alternative template. **(c)** This copies viral DNA into the host genome with rearrangements (16, 27).

HPV16 can drive cancer through abnormal episome replication without integrating into the host genome (33). Whether integrated or not, the virus hijacks the host’s DDR and repair machinery for viral replication. But integration often occurs and brings additional genomic instability and mutations that fuel cancer (34, 35). The interplay between HPV and the DDR is highly context-dependent, differing by replication versus transformation, episomal versus integrated status, lesion stage, and tumor type. During episomal replication, E6/E7 activate ATM/ATR and recruit BRCA1/RAD51 to viral foci, supporting genome amplification without significantly compromising host repair. Upon integration, sustained E6/E7 degrade p53/Rb, impair HR, and shift repair toward error-prone non-homologous end joining and alternative end joining, generating genomic rearrangements and copy-number alterations that mark the onset of HRD. Across lesion stages, low-grade lesions (predominantly episomal) exhibit minimal HRD, whereas high-grade lesions and invasive carcinomas show progressively higher integration rates and pronounced HRD, reflecting the stepwise accumulation of repair pathway disruptions as the lesion advances. Moreover, the degree of HRD varies across HPV-associated tumors: frequent in cervical cancer, moderate in head and neck squamous cell carcinoma, and intermediate in anal/penile cancers, likely reflecting differences in integration frequency and DDR gene mutational burdens at equivalent stages. Hence, DDR status should be assessed on a tumor-type and stage-specific basis, with lesion stage serving as a key stratification variable. HPV exerts dichotomous effects on the DDR: it activates repair pathways during episomal replication but drives HRD upon integration, with major implications for carcinogenesis and therapy. The main pathways involved are outlined below:

1. HR: The fidelity pathway bypassed (**Figure 2A**).
  Normal function: HR is an error-free way to repair DSBs. It operates in the S/G2 phases, using the sister chromatid as a template for faithful repair. Viral context: HR is not the mechanism through which HPV integrates into the host genome. HPV dirupts the host HR machinery in two ways. On one hand, through E7, it recruits HR factors like BRCA1 and RAD51 to viral episomes for accurate amplification. On the other, both E6 and E7 mislocalize these proteins away from cellular DNA breaks. In the episomal phase, HR co-option for viral replication compromises cellular DSB repair. This imbalance, upon integration, fuels genomic instability and favors error-prone integration.
2. NHEJ/microhomology-mediated end joining (MMEJ): Direct integration via end ligation (**Figure 2B, C**).
  Normal function: NHEJ (also classic NHEJ) and MMEJ (also alternative NHEJ) are primary DSB repair pathways in HPV-infected host cells. Because these pathways do not require a homologous template, they can operate throughout the cell cycle. NHEJ directly ligates broken ends, whereas MMEJ uses short microhomologies (5–25 bp), often causing deletions. Viral hijacking: Linear viral DNA is recognized as a “break end” by the host ligation machinery. In NHEJ, the Ku/DNA–PKcs complex binds viral and host ends, enabling ligation by DNA ligase 4/X-ray repair cross-complementing protein 4 (XRCC4), typically with minor insertions or deletions. In MMEJ, after limited resection by MRN/CtIP, viral microhomology anneals to host DNA. Repair is completed by DNA POLθ and DNA ligase 3/XRCC1, causing substantial host deletions at the junction.
3. Replication-dependent mechanisms (fork-stalling and template-switching [FoSTeS] and microhomology-mediated and break-induced replication [MMBIR]): Complex integration via template switching (**Figures 2D, E**).
  Normal origin: These are aberrant repair pathways that occur specifically at stalled or collapsed replication forks.
  Viral exploitation: In the MMBIR pathway, a collapsed replication fork generates a single−ended DSB which undergoes end resection. The exposed 3′ overhang invades viral DNA via microhomology to initiate replication. Repeated synthesis and template switching form complex chimeric rearrangements. Then the elongated strand reanneals to the original break end, resuming replication with viral sequences stably integrated into the host genome. In FoSTeS pathway, a stalled replication fork dissociates and invades adjacent viral DNA at microhomology to prime DNA synthesis. Following viral sequence replication, the nascent strand rejoins the original fork, leading to site−specific viral genome integration.

In summary, physiological DDR triggers transient cell-cycle arrest to facilitate DNA damage repair and preserve genomic integrity; by contrast, HPV hijacks host DDR signaling pathways to support viral genome amplification and drive permanent integration of viral DNA into the host genome. This integration leaves distinct mutational footprints: small indels for NHEJ, large deletions for MMEJ, and genomic rearrangements through FoSTeS/MMBIR (36). Notably, extrachromosomal DNA replication activates ATM-dependent DDR and depends on alternative NHEJ for maintenance, which indicates a targetable vulnerability in this kind of tumors (37). This mechanism mirrors episomal HR-HPV replication, suggesting that DDR-targeted therapies (e.g., ATM and POLθ inhibitors) currently under investigation for extrachromosomal DNA tumors may also benefit patients with HPV-related malignancies.

## TGF-β signaling at the crossroads of DDR, cell cycle progression, and HPV oncogenesis

More than 30 mammalian TGF-β superfamily members fall into TGF-β/nodal and bone morphogenetic protein (BMP) groups. The TGF-β/nodal group includes three TGF-β isoforms(TGF-β1,2,3), Nodal and its inhibitors (Lefty1/2), activin/inhibin system (four activins and one inhibin), and five growth differentiation factors. In contrast, the BMP group comprises eleven BMPs, four growth differentiation factors, and anti-Müllerian hormone (38). TGF-β signaling controls cell proliferation, plasticity, DNA repair, apoptosis, and migration, thereby modulating early development, tissue differentiation, wound healing, and immune regulation. But when it goes awry in certain cell settings, it fuels fibrosis, virus-driven inflammation, and cancer (39). This duality renders TGF-β paradox both attractive and challenging to target in immuno-oncology (40).

**Figure 3:**
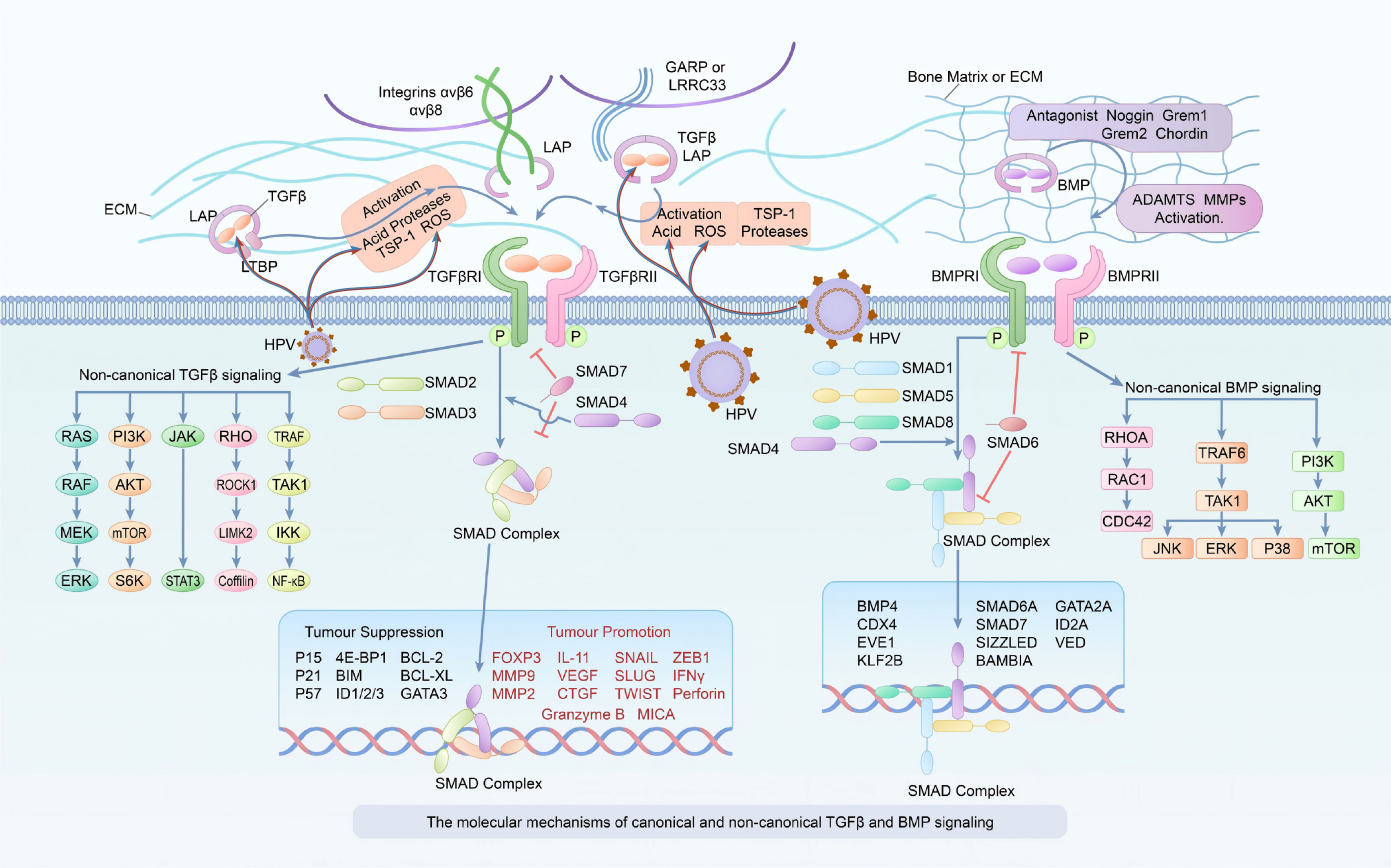
Canonical and non-canonical transforming growth factor β subgroup and bone morphogenetic protein subgroup signaling. TGF-β and BMP signals transduce through both canonical SMAD-dependent and noncanonical pathways. **(A)** TGF-β is secreted and stored as a latent complex with LAP in small latent complex (SLC), anchored to ECM via LTBPs or tethered to cell surface via GARP/LRRC33. Upon acidic pH, ROS, or TSP-1, TGF-β releases and activates; acidic pH and ROS are common in HPV-infected micro-environments. Active cytokine engages TGF-β RII/TGF-βRI heterotetramer, triggering TGF-βRI phosphorylation. In canonical TGF-β signaling, phosphorylated TGF-βRI phosphorylates SMAD2/3, which complex with SMAD4, translocate to nucleus together, and regulate tumor-suppressive/promoting gene transcription. SMAD7 recruits SMURFs to degrade TGF-βRI and dampen signaling. TGF-β also activates noncanonical cascades (e.g., RHO-ROCK, RAS-MAPK, TRAF6-TAK1, PI3K-AKT, JAK-STAT). TGF-β signaling is activated after DSBs, meanwhile, it positively regulates DSB repair and downstream β-catenin/PD-L1 pathway (38). **(B)** BMP activity is regulated by activators (e.g., ADAMTS, MMPs) and antagonists (e.g., Gremlin-1/2, Noggin, Chordin). Upon activation, BMPs bind receptors and phosphorylate SMAD1/5/8, which form SMAD1/5/8-SMAD4 complex, translocate to nucleus, and control differentiation, proliferation, and apoptosis genes. For attenuation, SMAD6 competes with SMAD1/5/8 for BMP type I receptor binding or promotes E3 ligase-mediated degradation of BMP type I receptors. BMPs also activate noncanonical pathways (e.g., RHOA-ROCK, TRAF6-NF-κB, PI3K-AKT) (38).

Endogenous TGF-β ligands are mostly homodimers, whereas heterodimers, (e.g., activin A/B and BMP9/10) are relatively uncommon. The canonical TGF-β signaling is initiated when a dimeric ligand binds sequentially to a pair of type II receptors (TGFBR2) and then to a pair of type I receptors (TGFBR1). This assembly triggers phosphorylation of a glycine/serine-rich (GS) motif within the type I receptor by the type II receptor kinase (38). This phosphorylation event displaces the inhibitory protein FKBP12 and activates the receptor kinase domain, which in turn phosphorylates specific receptor-regulated small mother against decapentaplegics (R-SMADs). In this process, the adaptor protein SMAD anchor for receptor activation (SARA) recruits cytoplasmic R-SMADs (SMAD2/3 for TGF-β, SMAD1/5/8 for BMPs) to the receptor complex. Phosphorylation of R-SMAD occurs at two C-terminal serine residues, creating a characteristic pSer-X-pSer motif. This modification enables phosphorylated R-SMADs to form stable heteromeric complexes with SMAD4. Despite lacking the phosphorylation motif, SMAD4 is essential to stabilize the complex and facilitate nuclear translocation (**Figure 2**). Structurally, SMAD proteins contain an N-terminal MH1 domain for DNA binding, a C-terminal MH2 domain for protein–protein interactions, and a central linker region. When the SMAD4-containing complex enters the nucleus, the MH1 domain binds specific SMAD-binding elements in DNA, ultimately leading to the modulation of target genes. Moreover, inhibitory SMADs (I-SMADs), which contain only a C-terminal MH2 domain, compete with R-SMADs for binding to activated TGFBR1. SMAD7 might inhibit TGF-β, BMP, and activin signaling by recruiting E3 ubiquitin ligases to SARA, SMAD4, R-SMADs, and TGFBR1, leading to proteasomal and lysosomal degradation. By contrast, SMAD6 preferentially antagonizes BMP signaling by blocking activation of SMAD1/5/8 and its interaction with SMAD4 (**Figure 3**) (38, 39).

TGF-β exerts dual roles in HPV-driven cancer: it initially suppresses viral gene expression and cell growth but is later co-opted by E6/E7 to promote transformation, angiogenesis, and immunosuppression (40). Mechanistically, HPV appears to upregulate autocrine TGF-β1 expression via C/EBPβ, although this event remains rarely reported and mechanistically unclear. By contrast, the downstream inhibition of TGF-β signaling through E7 is well established, which diverts the pathway from canonical tumor suppression toward non-canonical tumor promotion (41, 42). In high-grade lesions, HPV16 E5 was reported to promote cervical carcinogenesis through down-regulating TGFBR2 expression, and inhibit TGF-β/SMAD signaling, thereby disrupting epithelial homeostasis (43). E7 oncoproteins from both high- and low-risk HPV types directly bind SMAD1–4, blocking SMAD complex binding to target sequences and repressing transcription output (44). TGF-β1, TGFβR1, SMAD2, and SMAD3 are reportedly downregulated in cervical cancer compared with that in cervical intraepithelial neoplasia (CIN) (45). HPV oncoproteins E6 and E7, together with E5, may dysregulate the host cell cycle and delay epithelial differentiation to support viral replication. Furthermore, HPV E7 drives unscheduled S-phase entry by degrading Rb. This leads to constitutive E2F activation and S-phase gene expression. E6 works alongside E7: it promotes E6AP-mediated ubiquitin-dependent degradation of p53, thereby suppressing apoptosis and enabling viral persistence (46). HPV16 E7 binds to and inhibits SMAD2/3/4, potentiating immunosuppression of TGF-β1 (47). Autocrine or exogenous TGF-β1 triggers malignant transformation via telomere shortening and chromosomal instability in HPV16 E6/E7-immortalized cervical epithelial cells (48). In HPV-positive head and neck cancer, systemic inhibition of TGF-β signaling “unlocks” miR-182, leading to downregulation of BRCA1 and forkhead box O3. This shifts DNA repair from high-fidelity HR to error-prone alternative end joining, increases genomic instability, and ultimately sensitizes tumors to radiotherapy, cisplatin, and PARP inhibitors (49).

SMAD4 regulates most R-SMAD-mediated transcription. Loss of SMAD4 might increase genomic instability and cancer risk. This deficiency is present in at least 56% of HPV16-positive and 39% of HPV16-negative head and neck cancers, as well as 30% of pancreatic cancers and 40% of cervical cancers, and correlates with poorer progression-free survival (50). However, comparative analysis revealed significantly higher SMAD4 expression in HPV-positive tumors than in HPV-negative tumors in head and neck cancer (51). The tumor-suppressor BRCA1 upregulates Gadd45a to inhibit the E3 ligase β-Trcp1, thereby blocking ubiquitin-mediated SMAD4 degradation and maintaining TGF-β1-dependent growth control (52). SMAD4 directly binds to and stabilizes BRCA1 by blocking ARIH1-mediated ubiquitination; in pancreatic ductal adenocarcinoma, this promotes HR while suppressing error-prone NHEJ (53). These findings validate reciprocal stabilization between SMAD4 and BRCA1, which might be fundamental for TGF-β1-mediated tumor-suppression. Paradoxically, these findings also suggest aberrant SMAD4 as a potential therapeutic target in HPV-related cancers.

Intriguingly, reciprocal regulation between the TGF-β/epithelial-mesenchymal transition (EMT) and DDR/cell-cycle progression pathways potentially drives metastasis and therapy resistance (54). Cigarette smoke induces DNA DSBs, which activate the TGF-β and DDR pathways—marked by elevated TGF-β levels and phosphorylation of ATM, Chk2, γH2AX, and p53, with the most pronounced response occurring in S-phase cells (55). Ethanol induces oxidative DNA damage and activates the DDR in neural stem cells, promoting excision repair, a response significantly potentiated by the growth factor TGF-β1 (56). TGF-β signaling is potently activated after radiotherapy and chemotherapy, likely via the DDR and oxidative stress. In response to DNA damage, ATM phosphorylates and stabilizes c-Cbl, which in turn stabilizes the TGF-β type 2 receptor, thereby enhancing TGF-β signaling to induce cell cycle arrest during intestinal regeneration in mouse models (57). TGF-β1 pretreatment might accelerate the post-radiation DDR to promote cell survival via a SMAD2/3- and Ligase 4-dependent pathway, which is abrogated by the TGF-β receptor blocker SB431542 and siRNAs targeting SMAD2/3 or Ligase 4 (58). Blockade of the TGF-β pathway suppresses the expression of nucleotide excision repair genes, thereby exacerbating cisplatin-induced DNA damage and enhancing cancer cell death, which ultimately sensitizes breast cancer cells to chemotherapy (59). TGF-β/SMAD activation drives DDRs to confer radioresistance in glioma, and its inhibition by LY2109761 resensitizes glioma xenografts to radiation in mice (60). TGF-β also promotes the repair of bulky DNA lesions induced by polycyclic aromatic hydrocarbons and UV-C radiation via the SMAD4 pathway. This study was the first to show that TGF-β enhances nucleotide excision repair by facilitating the interaction and nuclear localization of key repair complexes such as ERCC1/XPA and ERCC1/XPF (61).

Collectively, these findings indicate that genotoxic stress in cancer triggers TGF-β signaling to promote DDR and that its inhibition increases therapeutic sensitivity. Although no drug specifically targeting the TGF-β/SMAD pathway has been globally approved for cancer treatment, the dual PD-L1/TGF-β targeting strategy remains biologically rational, given the complementary immunosuppressive roles of these two pathways. However, clinical translation has proven context-dependent. Bintrafusp alfa (M7824), the first bifunctional fusion protein of this class, was largely deprioritized by its original sponsors and is no longer listed in their active pipelines, although several U.S. National Cancer Institute-sponsored investigator-initiated trials are still ongoing. Relafusp alfa (SHR-1701/retlirafusp alfa) received approval from the National Medical Products Administration of China in January 2026 for first-line treatment of PD-L1-positive advanced gastric/gastroesophageal junction adenocarcinoma, yet its Phase III trial in cervical cancer was terminated and results in colorectal cancer have been disappointing. For HPV-associated cancers other than cervical, including HNSCC, evidence remains insufficient, and Phase II data are pending. The cervical cancer termination warrants caution, but should not be taken as definitive evidence against the entire class across all HPV-driven settings without biomarker-stratified analysis. Accordingly, at present, dual PD-L1/TGF-β blockade cannot be recommended as a standard approach in HPV-associated malignancies and should be restricted to well-designed biomarker-driven clinical trials.

## WNT/β-catenin pathway as a regulator of cancer cell stemness, therapy-resistance, and DDR in HPV-related cancer

Avitvation of β-catenin correlates with favorable prognosis in HPV-positive head and neck cancer but poor prognosis in cervical cancer (62). Under physiological conditions, cytoplasmic β-catenin is maintained at low levels through rapid degradation, whereas membrane β-catenin is linked to E-cadherin at adherens junctions (63). PP2A activates GSK3β through Ser9 dephosphorylation, enabling the destruction complex—comprising GSK3β, Axin1, casein kinase 1α (CK1α), and the tumor suppressor adenomatous polyposis coli (APC)—to regulate cytoplasmic β-catenin levels (64). Both HPV E7 and SV40 ST directly bind the PP2A catalytic subunit, leading to AKT–GSK-3β-mediated activation of Wnt signaling (65). Wnt signaling also determines β-catenin stability. In the absence of Wnt, phosphorylation by GSK-3β and CK1α triggers its degradation mediated by beta-transducin repeat-containing protein (β-TrCP). In contrast, active Wnt signaling inhibits GSK-3β through membrane-bound Dishevelled (Dvl) and phosphorylated low-density lipoprotein receptor-related proteins 5/6 (LRP5/6). This allows β-catenin to accumulate, enter the nucleus, and form a complex with T cell factor (TCF)/lymphoid enhancer factor (LEF) to activate target genes, such as c-Myc, vascular endothelial growth factor (VEGF), and cyclin D1 (66). HPV16 E6 may promote β-catenin stabilization and nuclear accumulation either via degrading Dlg1/Scribble to inactivate APC, impairing p53-SIAH-1-mediated degradation or facilitating NK2 Homeobox 1/Forkhead Box M1-mediated nuclear translocation (67). Moreover, it enhances E6AP function; E6AP stabilizes β-catenin by blocking GSK3β-mediated degradation, thereby facilitating nuclear translocation and β-catenin/TCF-driven transcription (68). HPV16 E6 was shown to activate Wnt11, Wnt3a, and Wnt7b/β-catenin signaling to fuel cervical cancer progression. The Wnt-inhibitory factor domain at the carboxyl terminus of WIF-1 and the netrin-like motif at the carboxyl terminus of secreted frizzled-related proteins inhibit Wnt signaling through Wnt protein binding. Therefore, targeting upstream Wnt regulators—such as porcupine inhibitors (e.g., LGK974) to block Wnt secretion, recombinant secreted frizzled-related protein decoys to sequester extracellular Wnts, or GSK3β activators to enhance β-catenin degradation—may inhibit HPV-driven Wnt/β-catenin signaling at the source.

A negative feedback loop exists between p53 and β-catenin. Activated β-catenin signaling induces expression of alternative reading frame (ARF), which activates p53 by inhibiting its negative regulator, mouse double minute 2 homolog (MDM2) (69). Concurrently, p53 promotes β-catenin degradation through the E3 ubiquitin ligase SIAH-1, thereby coordinating DDR and cell-cycle arrest (70). This p53–β-catenin balance might be disrupted by the HPV E6 oncoprotein, which degrades p53 and removes both the SIAH-1-mediated brake on β-catenin and p53-dependent genomic stability, resulting in β-catenin accumulation and genomic instability. The GSK3β–p53 interaction is critical for Wnt–DDR crosstalk. Diverse DNA-damaging agents induce DNA lesions to activate p53, which directly binds GSK3β for its activation and nuclear translocation, thereby augmenting p53 function and optimizing the Wnt signaling. GSK3β activation via p53 amplifies p53-mediated effects, elevates p21 and enhances caspase-3 activity to initiate DNA repair or apoptosis. Concurrently, this interaction limits GSK3β-mediated inactivation of β-catenin within the Wnt pathway, promoting p21 and caspase-3 upregulation during the cellular DDR (71). GSK3βphosphorylation of 53BP1 at T334 reprograms DNA repair from error-prone NHEJ to precise HR; importantly, targeting this GSK3B-53BP1 axis sensitizes tumors to PARP inhibitors independently of BRCA1 status (18, 72). Under normal conditions, β-catenin drives gene transcription as TCF/LEF co-activator, this is enhanced by poly(ADP-ribose) polymerase-1 (PARP-1) (73). After DNA damage, however, PARP-1 auto-poly(ADP-ribosyl)ation disrupts this interaction, and the Ku70/80 complex further suppresses transcription by displacing β-catenin from the complex (74). Wnt/β-catenin signaling interacts with MRN to repair of cisplatin-induced DNA interstrand cross-links. FH535 disrupts this repair and promotes cell survival, whereas CHIR99021 activates signaling and sensitizes cervical cancer cells to cisplatin, potentially enhancing cisplatin efficacy in cervical cancer (75).

Non-canonical Wnt signaling comprises two main branches: the Wnt/Ca^2+^ pathway and the Wnt/planar cell polarity (PCP) pathway. In the Wnt/Ca^2+^ branch, ligand–receptor binding activates G proteins and phospholipase C (PLC), triggering calcium release. In the Wnt/PCP branch, signaling through the ROR–FZD complex activates Dvl and small GTPases, such as Rho and Rac, which in turn stimulate ROCK and JNK. Taken together, these pathways regulate essential cellular processes, including motility, polarity, and gene transcription (76). Non-canonical Wnt signaling activates Yes-associated protein (YAP) and PDZ-binding motif (TAZ) through the non-canonical FZD/ROR/LATS1/2 pathway, thereby modulating gene programs involved in bone formation, cell migration, and Wnt feedback regulation (77). YAP/TAZ drive intestinal crypt regeneration and APC-loss overgrowth, and recruit β-TrCP to the destruction complex to degrade phosphorylated β-catenin in Wnt-OFF cells (78).

**Figure 4:**
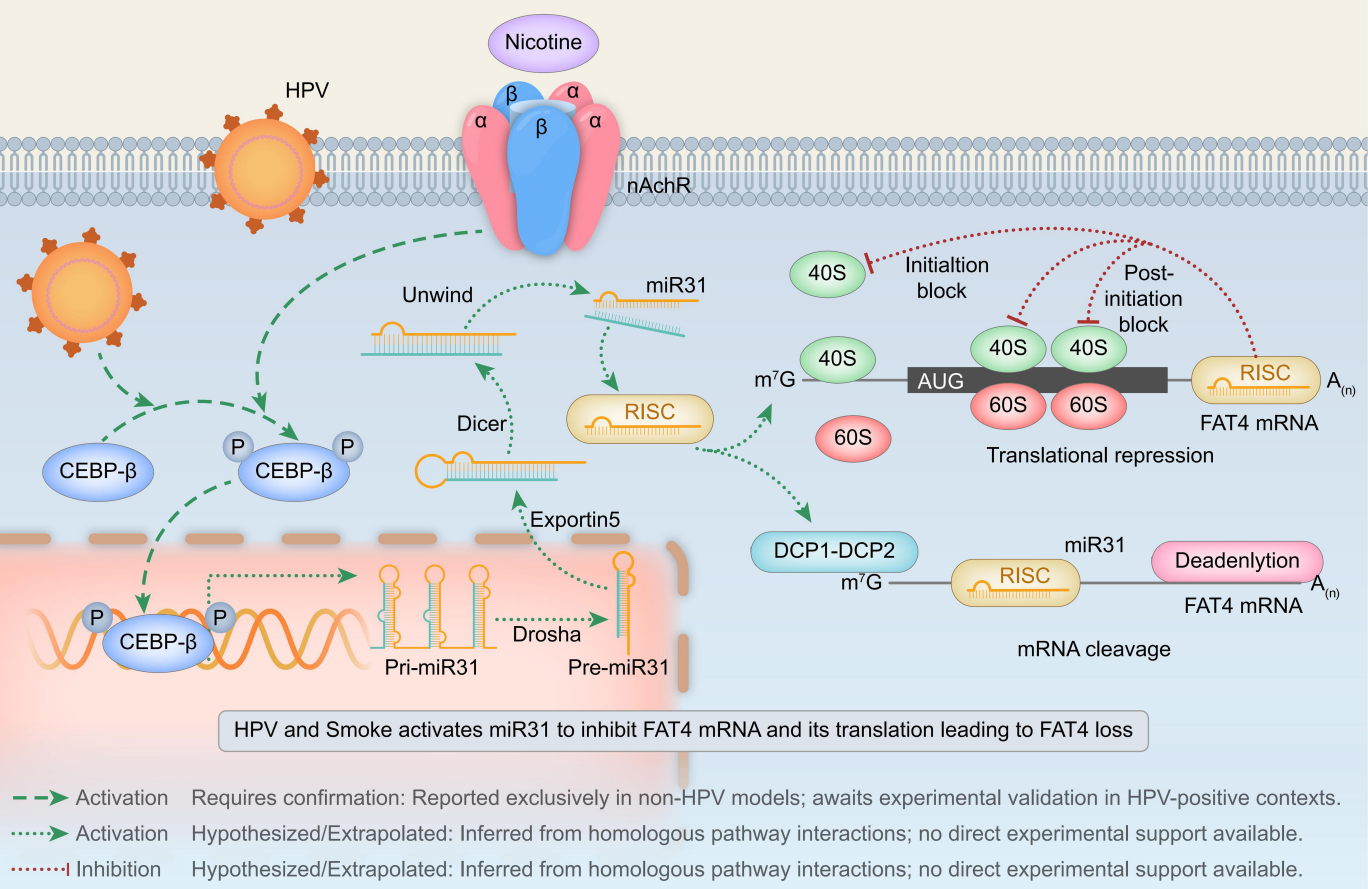
Putative molecular mechanisms linking HPV infection and cigarette smoke to FAT4 deficiency in cervical cancer. HPV and smoking may activate C/EBPβ, which translocates to the nucleus to upregulate pri-miR-31 transcription. Pri-miR-31 is processed by Drosha into pre-miR-31, exported via Exportin-5, and further cleaved by Dicer into mature miR-31. The single-stranded miR-31 is loaded into the RNA-induced silencing complex (miR-31-RISC). The RISC-bound miR-31 is predicted to bind FAT4 mRNA and guide mRNA degradation through deadenylation by the CCR4-NOT complex and decapping by DCP1–DCP2, followed by exonucleolytic digestion. In addition, miR-31-RISC may inhibit translation initiation or induce premature termination, thereby suppressing FAT4 expression at both the mRNA and protein levels. Of note, C/EBPβ activation by HPV and smoking remains to be validated in HPV-positive models, whereas the downstream miR-31-mediated FAT4 mRNA degradation and translational repression remain hypothetical (82, 83).

In HPV-negative head and neck cancer, abnormal β-catenin strongly correlates with loss of the tumor suppressor FAT Atypical Cadherin 1 (FAT1) (79) The cytoplasmic domain of FAT1 interacts with and sequesters β-catenin at the cell membrane, preventing nuclear entry (80). It also helps assemble a Hippo signaling complex that promotes YAP1 inactivation via phosphorylation. In HPV-positive tumors, FAT4 loss has been verified as a key regulator responsible for β-catenin activation and PD-L1-mediated immune evasion, although the mechanism underlying FAT4 downregulation remains elusive (81). Based on our ongoing and previous studies, we propose a putative model centered on the C/EBPβ/miR31/FAT4 axis (82, 83). Smoking may also activate this hypothetical axis, and FAT4 could be a potential therapeutic target and prognostic marker, but this model requires experimental confirmation in HPV-positive patients (**Figure 4**).

Beyond its transcriptional role, β-catenin functions as a signaling hub that integrates the TGF-β/PD-L1 signaling with the DNA damage response. In PD-L1-overexpressing cancer cells, β-catenin is stabilized by PD-L1 via PI3K/Akt and subsequently upregulates wild-type p53-induced phosphatase 1 (Wip1) by binding to its promoter. This axis promotes tumor progression *in vivo*, and indicates poor prognosis (84). By dephosphorylating Nemo-like kinase, Wip1 reduces LEF1 phosphorylation, promoting β-catenin/LEF1 complex formation and its activity (85). Current evidence indicates that β-catenin (regulating DNA ligase IV), Wip (dephosphorylating γH2AX), and β-catenin/TCF complex (regulating aldehyde dehydrogenase) converge to promote radio-resistance through enhanced DNA repair, outlining these players as potential biomarkers and sensitizing targets (86). In HPV-infected tissues, non-canonical TGF-β signaling activates β-catenin via SRC-mediated phosphorylation or GSK3β inactivation (87). Emerging evidence supports a direct crosstalk: TGF-β stimulation promotes β-catenin assembly with SMAD3/4 and Wnts secretion, stabilizing β-catenin and facilitating its nuclear translocation to cooperatively activate downstream target genes (88, 89). Conversely, Frizzled receptor and β-catenin modulates TGF-β signaling outputs. For example, the inhibitor ICG-001 diverts β-catenin from TCF to FOXO proteins, switching TGF-β activity from profibrotic to anti-inflammatory (90). Importantly, the Vasorin/Frizzled receptor inhibitor, Niclosamide, abolishes TGF-β/SMAD3-induced stabilization of β-catenin and PD-L1,therefore it has shown promise across diverse diseases, including cancer, infection, metabolic and inflammatory disorders (91).

HPV DNA replication-induced genomic instability may serve as the initial hit, whereas activation of the β-catenin/PD-L1 axis may act as the second hit that drives cervical cell transformation. The β-catenin/TCF transcriptional complex can be targeted through several strategies, including inhibitors that disrupt CBP/β-catenin binding (PRI-724), block β-catenin/TCF interaction (iCRT3/14), induce β-catenin degradation (MSAB/BC2059), or inhibit downstream coactivators (JQ1). Appropriate timing and combination of these drugs may suppress oncogenic transcription, reduce cancer stemness, and reverse the cold immune microenvironment in HPV-driven cancers. Targeting Wnt/β-catenin-driven cancer stem cells may benefit from combining Wnt pathway modulators with DDR inhibitors, as suggested by preclinical evidence (92). Although the efficacy of single-agent is limited, as evidenced by the failure of 9-ING-41 and PRI-724 in phase 2 trials, combining WNT/β-catenin inhibitors with immunotherapy, chemoradiotherapy, or DDR-targeting agents remains promising for clinical translation.

## PD-L1 as an immune modulator, DDR promoter, multifaceted biomarker, and compelling target in HPV-related cancer

Since its 1999 discovery as a T-cell immunity inhibitor, PD-L1 has become a broadly effective immunotherapy target across malignancies. PD-L1 expression has a context-dependent prognostic role in HPV-driven and associated HPV-negative cancers. In a meta-analysis of OPSCC cases, PD-L1 expression was detected in 57.14% and 64.28% HPV-negative and HPV-positive tumors (93). OPSCC PD-L1 positivity is common in younger, HPV-positive patients and predicts favorable disease free survival (94). Penile cancer PD-L1 positivity was 51.4% which correlated with advanced tumor stage, higher grade, lymph node involvement, and HPV positivity (95). PD-L1 positivity was reported in 72.7% of vulvar carcinoma, 61.1% of anal carcinoma, 91% of cervical squamous cell carcinoma, and 60% of usual cervical adenocarcinoma cases (96). Contrasting with OPSCC, elevated PD-L1 expression in anogenital and cervical carcinomas indicates unfavorable outcomes and correlates with advanced age, HPV negativity, and reduced overall survival (OS) (97). Thus, PD-L1 predicts diametrically opposite clinical outcomes depending on the tumor site, highlighting that its prognostic value is highly context-dependent and should not be generalized across HPV-related squamous cell carcinomas. Consistently, high HPV load in cervical cancer is associated with lower PD-L1 expression and poor prognosis.

**Figure 5:**
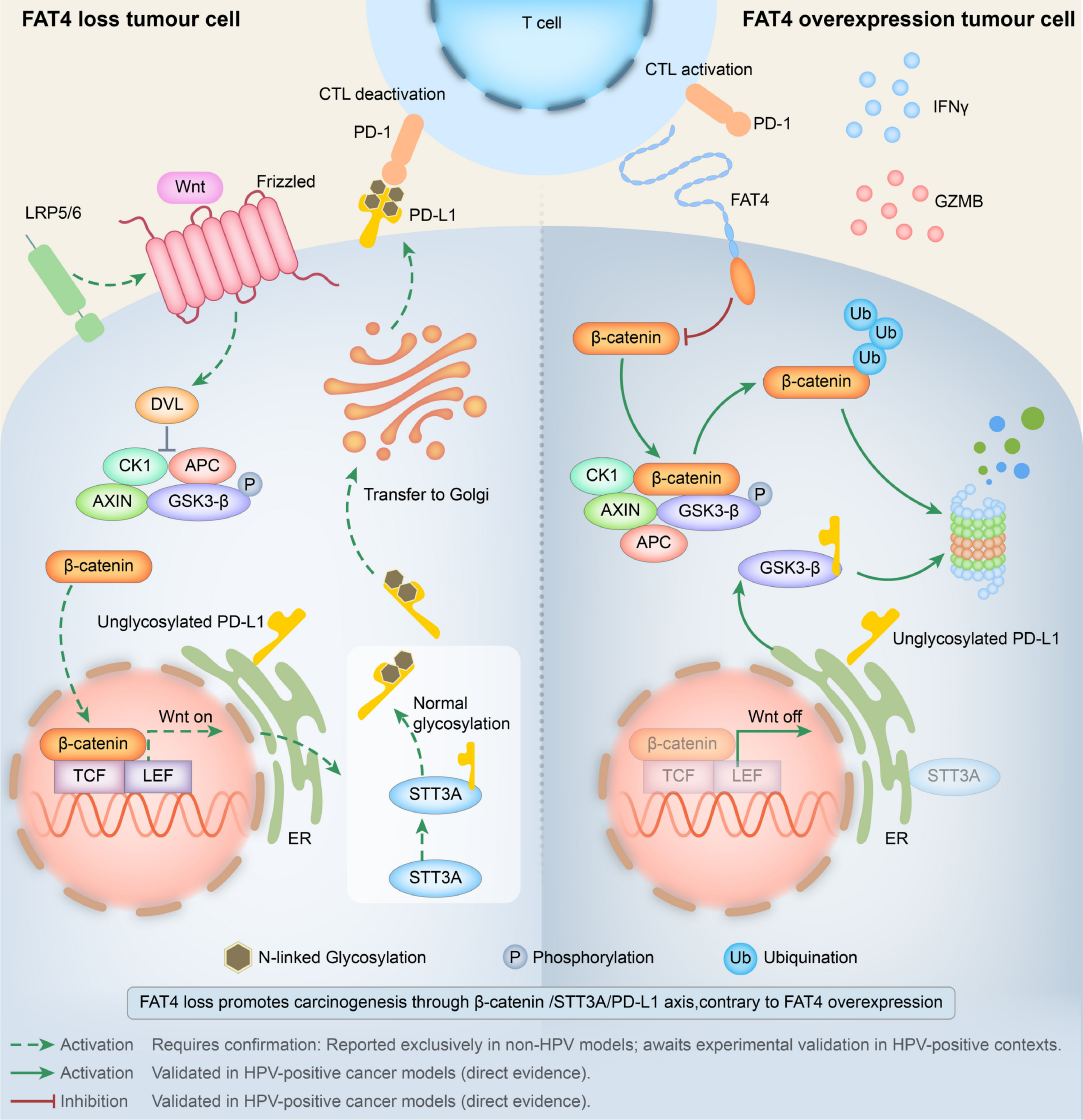
FAT4 loss promotes carcinogenesis through the β-catenin/STT3A/PD-L1 axis, which is counteracted by FAT4 overexpression. Left panel: In tumor cells with FAT4 loss, Wnt ligands binding to Frizzled/LRP5/6 activate canonical Wnt/β-catenin signaling. This recruits Dishevelled (DVL), which disrupts the CK1/Axin/APC/GSK-3β destruction complex, preventing β-catenin phosphorylation and degradation. Stabilized β-catenin translocates to the nucleus, binds TCF/LEF, and upregulates target genes, including cell-cycle regulators as well as STT3A and PD-L1. STT3A N-glycosylates PD-L1 via the N-glycan biosynthesis pathway, promoting ER-to-Golgi trafficking and membrane localization of PD-L1. These mechanisms have been reported exclusively in non-HPV models, and await experimental validation in HPV-positive contexts. Right panel: FAT4 exerts tumor-suppressive functions by binding β-catenin and sequestering it at cell junctions, thereby blocking nuclear translocation and facilitating GSK3β-mediated phosphorylation of β-catenin, which is subsequently degraded by the destruction complex. Consequently, FAT4 represses β-catenin-dependent transcription of PD-L1 and STT3A, leading to defective PD-L1 glycosylation and degradation, which in turn activates cytotoxic T lymphocyte (CTL)-mediated antitumor immunity. These mechanisms have been validated in HPV-positive cancer models (98, 146).

Mechanisms underlying PD-L1-mediated immune suppression have been extensively studied. Overexpressed FAT4 in cervical cancer engages β-catenin, restricts its nuclear translocation and facilitates destruction complex-mediated degradation. Blocked β-catenin signaling subsequently represses PD-L1 transcription and abrogates aberrant glycosylation of PD-L1 by STT3 oligosaccharyltransferase complex catalytic subunit A (STT3A), thereby enhancing anti-tumor immunity (98, 99). Loss of FAT4—a potential driver in numerous solid tumors, including carcinomas of the head and neck, cervix, lung, esophagus, prostate, ovary, liver, kidney, breast, brain, thyroid, and bile ducts —has been shown to fuel cervical and hepatocellular carcinomas (81, 100). However, the precise mechanisms responsible for FAT4 down-regulation remain elusive. Notably, etoposide decreases nuclear β-catenin and disrupts STT3APD-L1 axis, reducing PD-L1 expression and restoring the therapeutic efficacy of anti-Tim-3 therapy (**Figure 5**).

HPV E6/E7 upregulate PD-L1 by downregulating miR-142–5p or increasing HIF-1α expression and stability (101, 102). In HPV^+^ cervical cancer, Heat Shock Protein 90 and TGF-β upregulate PD-L1 via the Erb-B2 Receptor Tyrosine Kinase 2 (ERBB2)/phosphatidylinositol 3-kinase (PI3K)/AKT signaling cascade (103). HPV E6/E7 upregulate DNA topoisomerase I, which mediates DNA repair and activates cGAS to promote PD-L1-mediated immune evasion (104). Interferon-γ was reported to stimulate PD-L1 upregulation on extracellular vesicles in non-HPV-driven models such as metastatic melanoma, promoting tumor growth and immune suppression, its relevance in HPV-positive squamous cell carcinomas remains to be fully elucidated (105). Interferon-gamma inducible protein 16 promotes cervical cancer progression *in vitro* and *in vivo* through a signaling cascade initiated by the stimulator of interferon genes (STING), which proceeds through TANK-binding kinase 1 to activate Nuclear Factor κB (NF-κB)/PD-L1 expression (106). In HPV^+^ tumors, viral oncogenesis was shown to upregulate methyltransferase-like 3 (METTL3), which induces PD-L1 and fosters immunosuppression, correlating with poor immune infiltration and clinical outcomes (107). Mechanistically, METTL3 stabilizes PD-L1 mRNA through YTH N6-methyladenosine RNA binding protein (YTHDF) 1-mediated m6A modification, promoting cancer progression and CD8-positive T cell suppression across cancers (108, 109). Conversely, METTL3 can also degrade PD-L1 mRNA via YTHDF2, revealing context-dependent duality (110). Clinically, combined METTL3 inhibition and PD-1 blockade restore CD8^+^ T cell function in gastric cancer, highlighting therapeutic potential though requiring further validation (110).

CKLF-like MARVEL transmembrane domain-containing protein 6 (CMTM6) has emerged as a key regulator of both PD-L1 and β-catenin, exerting broad regulatory functions across multiple cancer types. CMTM6 binds these proteins at the plasma membrane and in recycling endosomes, preventing lysosomal degradation and stabilizing their expression (111, 112). Accordingly, CMTM6 depletion reduces PD-L1 levels and alleviates T-cell suppression, underscoring its potential as a therapeutic target. In colorectal cancer, CMTM6 and PD-L1 co-expression, accompanied by elevated β-catenin, predicts chemotherapy response (113). However, the clinical meanings of CMTM6-PD-L1 co-expression appear to be context dependent. Both markers positively correlate with β-catenin in head and neck squamous cell cancer (HNSCC); however, dependent on different context it indicates either poor prognosis or favorable survival accompanied by CD8+ T-cell infiltration. CMTM6/PD-L1 co-expression is also elevated in cervical cancer, indicating advanced disease and poor prognosis (114, 115). A newly developed antibody, H1A, has been shown to disrupt the PD-L1-CMTM6 interaction, promote PD-L1 degradation, activate innate and adaptive immunity, and achieve good antitumor efficacy in a humanized mouse model (115).

Accumulating evidence indicates that constitutive DNA damage upregulates PD-L1, which in turn promotes DNA damage repair and genomic stability. Consistently, in both 2D and 3D HNSCC models, irradiation induces the senescence markers p21 and γH2AX concurrently with PD-L1 and pERK1/2 upregulation (116). Building on the established role of PD-L1 in immune evasion through CMTM6-mediated cell-surface stabilization, a genome-wide CRISPR screening study identified PD-L1 as a key driver of cancer radioresistance. Mechanistically, irradiation induces PD-L1 N219 deglycosylation and CMTM6-dependent nuclear translocation, where the PD-L1 IgC domain directly interacts with Ku80 to stabilize its binding at DSB sites and accelerate NHEJ-mediated repair. This work reveals a non-canonical, immune-independent function of PD-L1 as a direct DNA repair regulator (117). Analysis of HNSCC and cervical cancer clinical samples shows that APOBEC3A expression and its mutational signature correlate with increased PD-L1 levels. Mechanistically, APOBEC3A induces replication-associated DNA damage, which activates the JNK/c-JUN pathway to upregulate PD-L1. Deficient mismatch repair may also contribute to PD-L1 upregulation through demethylation of the PD-L1 promoter in cervical cancer (118). Emerging data support a fundamental role for PD-L1 in DDR because of its intracellular and nuclear localization. In 2019, PD-L1 was initially reported to act as an RNA-binding protein that stabilizes more than 135 mRNAs encoding key DDR genes, including NBS1, BRCA1, ATM, POLQ, and FANCL, shielding them from RNA exosome-mediated degradation, thereby increasing cellular resistance to DNA damage (119). In 2022, tumor-intrinsic PD-L1 was shown to promote DDR by sustaining HR; it also suppresses cGAS–STING-mediated innate immune responses, and its depletion synergizes with DNA-PK inhibition to produce stronger synthetic lethality and antitumor immunity (120). A subsequent study showed that tumor-intrinsic PD-L1 regulates HR through interaction with BARD1, promoting BRCA1 nuclear accumulation and foci formation. Critically, genetic depletion of PD-L1, but not antibody blockade, induces synthetic lethality with PARP inhibitors and reverses olaparib resistance *in vivo* (121). Intracellular PD-L1 interferes with PIRH2 E3 ligase to stabilize CHK2, sustaining ATM-dependent HR repair. PD-L1 depletion leads to compensatory dependency on the ATR-CHK1 axis; subsequent CHK1 inhibition promotes error-prone NHEJ, sensitizing tumors to genotoxic therapies (122). Another study combined intracellular PD-L1 ablation with boron neutron capture therapy (BNCT) using 10B-loaded self-assembled nanoparticles that deliver PD-L1 siRNA. The siRNA dually inhibits DNA repair to enhance BNCT-mediated killing and blocks PD-L1 to trigger antitumor immunity, eliminating both primary and metastatic tumors (123).

Under stressful conditions in the tumor microenvironment, methylation inhibits Sirtuin-1 secretion, reducing its uptake by M2 macrophages and decreasing intracellular protein levels; this in turn upregulates PD-L1 expression on M2 macrophages and promotes CD8^+^ T-cell exhaustion (124). A study focused on type I interferon (IFN-I) revealed that PD-L1 exerts immune-independent functions that enhance cancer cell survival and DNA damage resistance through two mechanisms: suppression of the acute IFN-I response, which reduces DNA damage-induced cell killing, and maintenance of constitutive low-level IFN-β via the activated cGAS–STING pathway, which upregulates the IFN-related DNA damage resistance signature (IRDS) in cancer cells (125). Ionizing radiation upregulates PD-L1 in tumor-associated macrophages, and combination with ATM kinase inhibition enhances this effect via the STING/IFN-I/JAK-STAT pathway, boosting CD8^+^ T cell infiltration and antitumor efficacy *in vivo* (126). Immunotherapy resistance often arises from JAK mutations that reduce PD-L1 expression; however, DNA-damaging chemotherapeutics, including cisplatin and SN-38, can upregulate PD-L1 via the SRC–STAT1–IRF1 axis, indicating translational potential for improving immunotherapy efficacy (127). A nanomedicine with a PARP inhibitor, a photosensitizer, and a PD-L1-targeting peptide specifically recognizes PD-L1 high cancer cells and boosts PD-L1 expression. Photodynamic DNA damage plus PARP inhibition increases cytosolic DNA release, activates cGAS-STING pathway, and triggers systemic antitumor immunity that finally eliminate metastases (128). Notably, while these mechanistic and therapeutic insights have been largely obtained in non-HPV-driven models, their relevance in HPV+ tumors—where viral oncoproteins inherently perturb DDR pathways and PD-L1 expression—remains an open and intriguing question warranting dedicated investigation. HPV-positive HNSCC exhibits enhanced radiosensitivity and defective DSB repair; however, ATM and its downstream targets function normally, suggesting that a compromised ATM-orchestrated DDR network was the problem (129). HPV-positive HNSCC exhibits an immunologically “hot” phenotype, which is thought to result from sustained DNA damage, possibly attributable to a defective ATM axis (130).

Both ATR and PARP inhibitors suppressed HPV-positive cervical cancer cells as single agents. Sequential treatment (ATR inhibitor first, then PARP inhibitor) was synergistic. The former raised PARP expression and increased reliance on PARP-driven repair, while the latter contributed to significant tumor control *in vivo*. ATR inhibition has also been reported to enhance radiotherapy response across HNSCC preclinical models with differing HPV status (131). The nanoadjuvant Tpp-Met@MnO@Alb overcomes radioimmunotherapy resistance through multiple components: the MnO2 core, which alleviates tumor hypoxia and generates Mn^2+^ to activate the cGAS–STING pathway, and conjugated Tpp-Met, which simultaneously inhibits PD-L1 and TGF-β1. This multitarget strategy effectively overcomes therapeutic resistance, suppresses local and metastatic tumor growth, and establishes durable antitumor immunity (132). PD-L1 was shown to form complex with other factors at GAS6, EGR1, and its own gene promoters, thereby creating a self-reinforcing transcriptional circuit in cancer cells (133).Since these findings were obtained in a non-HPV model, their applicability to HPV^+^ tumors, where viral oncoproteins constitutively disrupt DDR and immune homeostasis, remains unclear.

Current clinical guidelines have integrated immune checkpoint inhibitors (ICIs) into treatment paradigms for HNSCC (including OPSCC) and cervical cancer, based on pivotal phase III trials: KEYNOTE-048 (NCT02358031), COMPASSION-16 (NCT04868708), and KEYNOTE-826 (NCT03635567) validated pembrolizumab as first-line treatment for recurrent/metastatic HNSCC. Pembrolizumab monotherapy is indicated for patients with PD-L1 CPS ≥ 1, with maximal benefit observed among those with CPS ≥ 20, whereas pembrolizumab combined with cisplatin/5-fluorouracil platinum-based chemotherapy remains effective regardless of PD-L1 status. For locally advanced cervical cancer, ICIs are combined with concurrent chemoradiation in patients with PD-L1 CPS ≥ 1. For metastatic disease, ICIs are paired with chemotherapy, with or without bevacizumab, typically independently of PD-L1 expression. In June 2025, the U.S. Food and Drug Administration approved perioperative pembrolizumab for resectable locally advanced HNSCC in patients with PD-L1 CPS ≥ 1. Supported by the event-free survival benefit observed in KEYNOTE-689, this approval establishes a new standard of care for neoadjuvant/adjuvant immunotherapy combined with surgery and radiotherapy (134). However, ICI efficacy is limited by resistance and immune-related adverse events (irAEs). Most strategies discussed here aim to overcome resistance and enhance efficacy. Although PD-1/PD-L1 inhibitors are generally better tolerated than other therapies, irAEs commonly affect the skin (pruritus), gut (diarrhea and colitis), and endocrine organs (135). Notably, irAE profiles may be different by HPV status; for example, HPV^+^ HNSCC patients receiving ICIs may have distinct irAE patterns due to enhanced immune infiltration, requiring tailored monitoring. The primary resistance appears to be pathway-specific. In HPV-negative disease, intrinsic mechanisms such as copy-number alterations (e.g., 9p loss) or signaling bypass (e.g., the AXL-PI3K-PD-L1 axis) are recognized contributors. However, for HPV-positive tumors, recent evidence suggests that the Dsg2/miR-146a/IL-8 signaling axis, may play a key role in shaping ICI resistance. In these cases, the resistance is linked to specific non-canonical pathways rather than a generalized T-cell dysfunction (136).

HPV-positive tumors are particularly vulnerable to DDR and PD-L1 blockade, as E6/E7 oncoproteins simultaneously drive genomic instability and PD-L1 upregulation—a dual dependency amenable to therapeutic exploitation. Preclinical PD-L1-targeting strategies (bispecific antibodies, nanoparticles, oligonucleotides) aim to trigger immunogenic cell death and reverse immunosuppression (137); newer modalities target PD-L1 transcription, glycosylation, or stability via PROTAC degraders (138). Bispecific antibodies co-targeting PD-L1 with 4-1BB, LAG-3, TIGIT, TGF-β1, VEGF, or CD47 are also being pursued (139). Critically, combining genotoxic agents with PD-L1 and DDR inhibitors may disrupt repair and enhance immunogenicity in HPV^+^ tumors, albeit with increased toxicity risk (140). However, biomarker-driven patient selection remains key to optimizing these combinatorial approaches.

## Discussions and conclusions

Surgery is the standard of care for most high-grade CIN and selected early-stage HPV-related cancers, particularly cervical and vulvar cancers. However, for HPV-related oropharyngeal and anal cancers, definitive radiotherapy or chemoradiotherapy plays an equally important or dominant role. For penile cancer, surgery is the standard for early stages, neoadjuvant chemotherapy plus surgery for locally advanced disease, with a limited role for definitive radiation (141). Despite significant advances in organ preservation and adjuvant therapies, clinical outcomes in HPV-associated OPSCC and anogenital cancer are not always satisfactory. To translate our mechanistic framework into clinically accessible terms, **Table 3** organizes the major therapeutic modalities for HPV-associated cancers by disease stage and regulatory/developmental status, distinguishing approved regimens from investigational strategies, and provides standardized evidence grading (FDA-approved, NCCN-recommended, Phase I/II, and preclinical/hypothetical) for each entry (142). Although this review discusses multiple intervention strategies, readers should recognize that the levels of evidence vary considerably across modalities. Our aim is to bridge fundamental biology and clinical trial design, not to supplant current practice guidelines. All discussion of unapproved agents is intended solely to inform the rationale for future investigational protocols.

**Table 3:** Clinical intervention strategies in HPV-associated cancers by disease setting.

Disease setting	Approved / standard-of-care	Investigational / emerging (with evidence level)
Precancer / early-stage	Surgical excision (LEEP, conization) - *standard*	Therapeutic HPV vaccines (Phase I/II, mixed results)
(CIN, early cervical, early HNSCC)	PDT - *investigational (Phase II)*	Topical antivirals (Phase I/II)
Locally advanced	Chemoradiation (cisplatin-based) - standard	Neoadjuvant immunotherapy (Phase II)
(cervical, HNSCC)	Pembrolizumab + chemoradiation for cervical (FDA-approved 2024)	Vaccine + ICI combinations (Phase I/II)
Recurrent / metastatic	Cervical: Pembrolizumab +chemotbevacizumab (1st line), Tisotumab vedotin (2nd line), Cemiplimab (2nd) - *FDA approved*	ICI + therapeutic vaccine combos (Phase II)
(cervical, HNSCC)	HNSCC: Pembrolizumab (CPS≥1) - *FDA approved*	DDR inhibitors (preclinical / hypothetical)
		TGF-B/ß-catenin/PD-L1 targeting (preclinical / hypothetical)
Other HPV-related sites	Anal: Chemoradiation (5-FU/mitomycin) - *standard*; Retifanlimab+chemo (1st line metastatic, FDA-approved 2025); Pembrolizumab / Nivolumab (2nd line) - *NCCN category 2A*	Anal: ICI combinations (Phase II)
(anal, vulvar, penile, vaginal)	Vulvar: Surgery (primary) - *standard*; Chemoradiation for locally advanced/unresectable - *standard*; Carboplatin (category 2A) as chemoradiation alternative if cisplatin unavailable; Ipilimumab + Nivolumab (2nd line recurrent/metastatic) - *NCCN category 2A*	Vulvar: Pembrolizumab + chemoradiation (unresectable, Phase II); Fam-trastuzumab deruxtecan for HER2-positive (NCCN category 2A); Lenvatinib + Pembrolizumab (Phase II); TROP2/TF/NECTIN4 ADCs (preclinical)
	Penile: Surgery (organ-sparing or radical) - standard; Topical 5-FU or imiquimod for superficial lesions - *standard*; Radiotherapy / brachytherapy for select cases - *standard*; Platinum-based chemotherapy for locally advanced/metastatic - *standard*	Penile: Cemiplimab + chemotherapy (Phase II, EPIC-A trial); Avelumab (Phase II, ALPACA trial, clinical benefit rate 21%); Niraparib + anti-PD1 (preclinical); Pembrolizumab + platinum-based chemotherapy (under NCCN review); DDR inhibitors / targeted agents (preclinical)

Evidence categories (footnote): • Standard = NCCN category 1 or established clinical practice. • FDA - approved = approved for the specific indication • NCCN category 2A = recommended by NCCN based on lower-level evidence, with uniform concensus. • Phase I/II active clinical trials, no conclusice efficacy data. • Preclinical / hypothetical = only cell/animal data or biological extrapolation; requires further validation.

Smoking prolongs HPV infection, worsens treatment response, and elevates postoperative second cancer risk (143–145). Mechanistically, it may activate TGF-β-β-catenin-PD-L1 via C/EBPβ/miR-31/FAT4, promoting immune evasion and malignant progression (98, 146). Smoking cessation should complement vaccination and screening in HPV-related cancer prevention (147). Finally, as suggested, we have added a summary **Table 4** that lists the pathway components, HPV-related cancer types, evidence types, therapeutic implications, and evidence strength for each node in the DDR-TGF-β-β-catenin-PD-L1 axis (148),and **Table 5**, which outlines the clinical translation challenges including toxicity, resistance mechanisms, and biomarker development for each therapeutic strategy (149, 150).

**Table 4:** Mechanistic pathway, evidence grading, and therapeutic implications in HPV-driven cancers.

Pathway component	HPV-related cancer type(s)	Evidence type	Therapeutic implication	Evidence strength
DDR activation (ATM/ATR)	Cervical, HNSCC	Directly validated in HPV-positive cell lines and tissues (E6/E7-mediated activation)	Potential target for viral replication inhibition; enhances sensitivity to genotoxic agents	Strong
BRCA1/RAD51 hijacking	Cervical (episomal phase)	Directly validated in HPV-positive models (viral replication foci recruitment)	Exploitation of HR machinery for viral persistence; PARPi sensitivity context-dependent	Moderate (phase-dependent)
HRD & shift to error-prone end-joining	Cervical (integrated phase), HNSCC	Reported in HPV-positive tumors (genomic scar signatures); functional validation in cell lines	Synthetic lethality with PARP inhibitors; rational combination with DNA-damaging agents	Moderate
TGF-β pathway suppression	Cervical, HNSCC, anal	Directly validated in HPV-positive models (E6/E7-mediated downregulation of TGF-BR signaling)	TGF-β blockade (e.g., galunisertib) to restore anti-tumor immunity; dual PD-L1/TGF-β bispecifics (investigational)	Strong (mechanism); Weak (clinical in HPV)
β-catenin / WNT activation	Cervical, HNSCC	Directly validated in HPV-positive samples (nuclear B-catenin accumulation); linked to FAT4 downregulation	β-catenin inhibitors (preclinical); potential to reverse immune evasion and stemness	Moderate
PD-L1 upregulation	Cervical, HNSCC, anal, vulvar	Directly validated in HPV-positive tumors (E6/E7 - B-catenin - PD-L1); correlates with CPS score	Anti-PD-1/PD-L1 immunotherapies (FDA-approved in multiple settings)	Strong (clinical)
FAT4/C/EBP-β/miR-31 axis	HNSCC (hypothesized), cervical (speculative)	Reported in non-HPV models (lung, breast); extrapolated from pathway biology; no direct HPV+ patient validation	Smoking-related biomarker; potential epigenetic target (hypothetical)	Weak / Hypothetical
Dual PD-L1 TGF-β bispecifics (bintrafusp alfa, relafusp alfa)	Cervical (terminated Phase III), HNSCC (Phase II ongoing), gastric (approved)	Direct clinical trial data available, but negative in cervical; positive in gastric (NMPA approval 2026)	Restricted to biomarker-driven trials; not recommended as standard in HPV-related cancers	Weak (HPV-specific negative data)

Evidence Strength Definition: Strong = validated in HPV- positive patient samples or isogenic cell models with functional assays; Moderate = supported by HPV - positive models but with contexual limitations (e.g., phase- dependent, tumor- type specific); Weak = reported in non- HPV systems or early-phase clinical data without HPV- specific confirmation; Hypothetical = extrapolated form general pathway biology with no direct HPV evidence. All investigational/preclinical implications are presented solely to inform future research and do not constitute clinical recommendations.

**Table 5:** Clinical translation challenges of therapeutic strategies targeting the TGF---β/β-catenin/PD-L1-DDR axis in HPV-driven cancers.

Pathway omponent	Toxicity	Resistance mechanisms	Biomarker development
DDR activation (ATM/ATR)	Myelosuppression, GI toxicity (ATR inhibitors)	Replication fork stabilization, ATR overexpression	γH2AX foci, p-ATM/p-ATR levels (investigational)
BRCA1/RAD51 hijacking	Bone marrow suppression, fatigue	(PARPi)PARP1 reversion mutations, 53BP1 loss	HRD genomic scar signatures (limited in HPV)
HRD & error-prone end- joining	Similar to PARPi; additive with DNA damage agents	Reversion mutations in BRCA1/2, RAD51 upregulation	Genomic instability indices (investigational)
TGF-β pathway suppression	Cardiotoxicity, skin lesions, GI bleeding	TGF-β isoform redundancy, alternative SMAD pathways	Plasma TGF-β levels, SMAD phosphorylation (not validated)
ß-catenin / WNT activation	Osteotoxicity (bone density loss), GI toxicity	ß-catenin mutations, alternative WNT ligand secretion	Nuclear β-catenin IHC, AXIN2/LEF1 expression (investigational)
PD-L1 upregulation	Immune-related adverse events (pneumonitis, colitis, dermatitis)	IFN-γ pathway defects, JAK1/2 mutations, PD-L2 upregulation, Treg infiltration	CPS/TPS score (validated but HPV-specific thresholds unclear)
FAT4 / C/EBP-β / miR-31 axis	Unknown (preclinical)	Unknown	FAT4 methylation, miR-31 expression (exploratory)
Dual PD-L1/TGF-β bispecifics	Bleeding, keratoacanthoma, skin toxicity	TGF-β pathway escape, alternative immune checkpoints	No identified predictor in biomarker-negative cervical cohort

In conclusion, HPV-related cancer management varies by site and stage: CIN is managed by surgery, with PDT or topical agents as alternatives in select cases; early cervical/vulvar invasive cancers are primarily surgical; oropharyngeal/anal and locally advanced cervical/vulvar cancers are chemoradiotherapy-dominant; whereas locally advanced penile cancer requires neoadjuvant chemotherapy followed by surgery. Novel targeted and antiviral therapies represent the next frontier.

There are some limitations of this review to be addressed in the future. First, most studies on TGF-β/β-catenin/PD-L1-DDR crosstalk in HPV-related cancers relies on *in vitro* models, with insufficient animal or clinical sample studies, weakening the confidence in findings. Second, late-phase trials targeted TGF-β/β-catenin/PD-L1-DDR axis in HPV-related cancer are few, hindering therapy assessment and clinical translation.

To address these gaps, future HPV-related cancer research should focus on:

- Prioritizing combinations of TGF-β/β-catenin/PD-L1 inhibitors with genotoxic therapies or DNA repair inhibitors for multipathway tumor suppression and
- Designing HPV-specific Phase II/III trials incorporating biomarker-driven patient selection to validate combination therapy efficacy and accelerate clinical translation.

## Glossary

APOBEC3A: Apolipoprotein B mRNA editing enzyme catalytic subunit 3A
ARIH1: Ariadne RBR E3 Ubiquitin Protein Ligase 1
AXL-PI3K: AXL receptor tyrosine kinase –
BRCA1: Breast cancer type 1 susceptibility protein
cGAS-STING: Cyclic GMP-AMP synthase –
CD47: Cluster of differentiation 47
ERBB2: Erb-B2 receptor tyrosine kinase 2 (HER2)
ERK1/2: Extracellular signal-regulated kinase 1/2
FANCL: Fanconi anemia group L protein
FKBP12: FK506-Binding Protein 12
FOXO: FOXO, Forkhead Box O
FZD/ROR/LATS1/2: Frizzled/Receptor tyrosine kinase-like orphan receptor/Large tumor suppressor kinase 1/2 (Hippo pathway components)
H2AX: H2A histone family member X
ICI: Immune checkpoint inhibitor
JAK-STAT: Janus kinase –
JNK: c-Jun N-terminal kinase
LAG-3: Lymphocyte activation gene 3 protein
METTL3: Methyltransferase-like 3
NBS1: Nijmegen breakage syndrome protein 1
RAD51: DNA repair protein RAD51
ROCK: Rho-associated coiled-coil containing protein kinase
PP2A: Protein Phosphatase 2A
PROTAC: Proteolysis-Targeting Chimera
SIAH-1: Seven in absentia homolog 1
TCF/LEF: T-Cell Factor/Lymphoid Enhancer-Binding Factor
TIGIT: T cell immunoreceptor with Ig and ITIM domains
TOPBP1: DNA Topoisomerase II Binding Protein 1
VEGF: Vascular Endothelial Growth Factor
WRN: Werner Syndrome RecQ-like Helicase
